# Complementary and alternative therapies for post-traumatic stress disorder: A protocol for systematic review and network meta-analysis

**DOI:** 10.1097/MD.0000000000021142

**Published:** 2020-07-10

**Authors:** Kai Song, Fanjie Xiong, Ning Ding, Ailing Huang, Hong Zhang

**Affiliations:** a College of Acupuncture and Tuina; b College of Clinical Medicine, Chengdu University of Traditional Chinese Medicine, Chengdu, Sichuan Province, China.

**Keywords:** complementary and alternative therapies, protocol, post-traumatic stress disorder, systematic review

## Abstract

**Background:**

Post-traumatic stress disorder (PTSD) is a psychiatric disorder. While bringing psychologic pain to patients, it also damages their social function, which is a great threat to people's life and health. Complementary and alternative medicine (CAM) therapies have been used clinically to treat PTSD; however, the selection strategies of different CAM interventions in clinical practice is still uncertain, and the purpose of this study is to evaluate the efficacy and acceptability of different CAM therapies using systematic review and network meta-analysis.

**Methods:**

According to the strategy, the authors will retrieve a total of 7 electronic databases by June 2020. After a series of screening, the 2 researchers will use Aggregate Data Drug Information System and Stata software to analyze the data extracted from randomized controlled trials of CAM therapies for the PTSD. Finally, the evidence grade of the results will be evaluated.

**Results:**

This study will provide a reliable evidence for the selection of CAM therapies for PTSD.

**Conclusion:**

The results of this study will provide references for evaluating the influence of different CAM therapies for PTSD, and provide decision-making references for clinical research.

## 1 Introduction

Post-traumatic stress disorder (PTSD) is a debilitating mental disorder that develops following direct or indirect exposure to an extremely stressful (traumatic) event or series of events, such as war, sexual violence, and life-threatening accidents.^[1–3]^ It is characterized by 3 symptom clusters: re-experiencing the traumatic event, avoidance, and hyperarousal.^[4]^ Diagnostic and Statistical Manual of Mental Disorders (DSM-IV) classified PTSD as anxiety disorder,^[5]^ and it is divided into 3 types: acute (symptoms lasting for <3 months), chronic (symptoms lasting for at least 3 months), with delayed onset (symptoms appearing at least 6 months after the stress). Present research suggests that the pathogenesis of PTSD is related to multiple neurobiologic systems, especially serotonergic (5-HT), noradrenalinergic, and neuropeptidergic systems, as well as the hypothalamic-pituitary-adrenal axis.^[6]^ There is no doubt that PTSD can increase psychologic distress, and severely affect patients’ quality of life and social stability. Epidemiologic studies^[7–9]^ have revealed that more than half of the world's population experience stressful events, about 25% and 30% of people who experience a traumatic event may develop PTSD, and the lifetime and average prevalence of PTSD are 6.8% and 8%, respectively. PTSD is also comorbid with depression, anxiety disorders and substance abuse,^[10]^ resulting in a severe financial burden.

Currently, the typical treatment methods for patients with PTSD are pharmacologic and psychologic interventions, for example, cognitive behavior therapy and antidepressant drugs.^[11,12]^ And among the antidepressants, selective serotonin reuptake inhibitors, such as sertraline, are the strongest current evidence base for pharmacologic treatment.^[13]^ However, due to significant individual differences and side effects, a large portion of the patients cannot obtain satisfactory treatment response.^[14]^ Therefore, better treatment strategies is an urgent need in PTSD.

Complementary and alternative medicine (CAM), generally refers to techniques that are integrated with or substituted for traditional practices of western medicine.^[15]^ CAM interventions include a range of therapies, from yoga to acupuncture to neurostimulation, these modalities are becoming more widespread around the world.^[16]^ In recent years, CAM approaches have been used clinically to treat a variety of mental health disorders, including PTSD.^[17]^ According to a study, about 21% of CAM users met diagnostic criteria for at least 1 problematic mental disorder.^[18]^ An increasing number of researchers are studying the use of CAM approaches for treating PTSD, include acupuncture, moxibustion, Chinese herbal medicines, meditation, yoga, deep-breathing exercises, progressive relaxation, and tai chi.^[19]^ Kim et al^[20]^ reviewed the literature and found support for beneficial effect of CAM interventions on symptoms of PTSD.

There are several CAM therapies for PTSD and their efficacy has been assessed by some systematic reviews. However, there has been no network meta-analysis (NMA) of the differences between different CAM therapies for PTSD. The aim of this study is to assess efficacy and acceptability of different CAM therapies, and to provide a clinically useful reference of the comparative evidence that can be used to guide decisions about treatment of PTSD.

## 2 Methods

### 2.1 Protocol and registration

This protocol follows the Preferred Reporting Items for Systematic Reviews and Meta-Analyses Protocols (PRISMA-P) guidelines.^[21]^ The NMA protocol has been registered on Open Science Framework platform (https://osf.io/42rdz/), registration number: DOI 10.17605/OSF.IO/42RDZ.

### 2.2 Ethics

Since NMA does not involve the collection of private information, this research does not require ethical approval.

### 2.3 Eligibility criteria

The participant (P), intervention (I), comparator (C), outcome (O), and study design (S) are the 5 main factors determining the inclusion and exclusion criteria of this research.

#### 2.3.1 Type of participant

All studies including patients with PTSD diagnosed by any set of criteria were eligible for inclusion, such as DSM-5, International Classification of Diseases (ICD-10), regardless of gender, age, educational background, nationality, or outpatient therapy or inpatient therapy.

#### 2.3.2 Type of interventions and comparators

Complementary and alternative therapies for treating PTSD include acupuncture, moxibustion, Chinese herbal medicines, meditation, yoga, deep-breathing exercises, mind-body therapy, and tai chi. These interventions can be used alone or in combination. Controlled interventions included control groups with no treatment, sham/placebo groups, or other conventional treatments.

#### 2.3.3 Type of outcomes

##### 2.3.3.1 Primary outcomes

The main outcome has to be measured by scores on a standardized, observer-rated instrument, for example, the Clinician-Administered PTSD Scale for DSM-IV (CAPS),^[22]^ or a validated self-report measure of PTSD symptoms, for example, the Posttraumatic Stress Disorder Checklist-DSM-5 Version (PCL-5).^[23]^

##### 2.3.3.2 Secondary outcomes

1. Depression and anxiety score measured by standardized scale, for example, Hamilton depression scale^[24]^ and Hamilton anxiety scale.^[25]^
2. Sleep quality parameters extracted from sleep scale, for example, sleep quality index.^[26]^
3. Quality of life obtained from the corresponding scale.
4. Adverse events may be taken into consideration.

#### 2.3.4 Study design

This study is a systematic review with NMA of RCTs on complementary and alternative therapies for the PTSD. All relevant RCTs using CAM therapies for the PTSD will be included. Quasi-RCTs, duplications, animal trails, review documents, clinical experience, and case reports will be excluded. Additionally, only English and Chinese literature will be search for this study.

### 2.4 Literature retrieval strategy

Computer retrieval of published RCTs of complementary alternative therapy for PTSD is conducted in PubMed, the Cochrane Library (issue 6, 2020), Embase, China National Knowledge Infrastructure, China biological medicine, Chongqing VIP, and Wan-fang databases. The time limit of document retrieval is from the establishment of each database to June 30, 2020. The language is limited to English and Chinese. In addition, inclusive literature from the field and references from previous evaluations will be manually retrieved to find other potentially relevant articles. Chinese search terms mainly include: “post-traumatic stress disorder”; English search words include “Post-traumatic stress disorder,” “PTSD,” “acupuncture,” “moxibustion,” “Chinese herbal medicines,” “meditation,” “Yoga,” “deep-breathing exercises,” “mind-body therapy,” and “Tai Chi.” Taking PubMed as an example, the initial retrieval strategy is shown in Table 1 and will be adjusted according to the specific database.

**Table 1:**
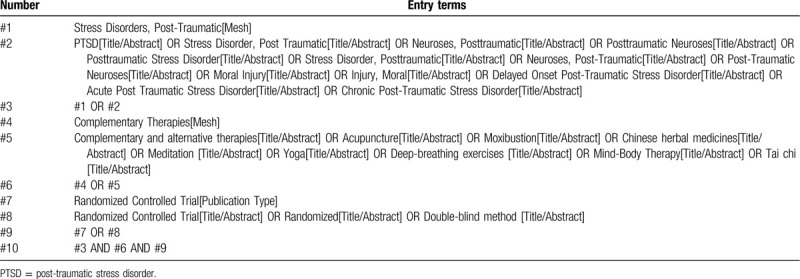
PubMed search strategy.

### 2.5 Literature selection and data extraction

**Figure 1:**
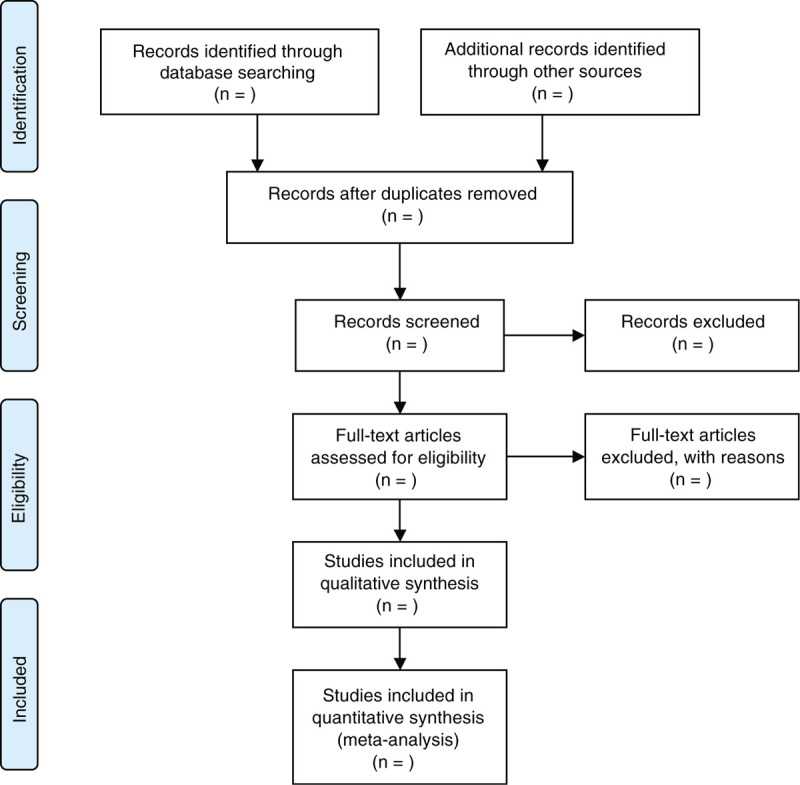
Flow chart of literature screening.

The study selection program will follow the PRISMA guidelines. As shown in Figure 1, Kai Song and Fanjie Xiong will independently screen literatures according to inclusion and exclusion criteria: The retrieved literatures will be imported into Endnote X9 software for rechecking, and duplicate references are removed; By reading the title and preliminarily screening the abstract, exclude the literature that obviously does not meet the inclusion criteria; Download and read the full text for rescreening; After the final inclusion, the predesigned data extraction table is used for data extraction, and the results will be cross-checked; If there is any disagreement, the 3rd researcher Ailing Huang will be asked to assist in the judgment. The main content of data extraction includes: basic information of literature (title, journal, author, publication date), basic situation of the research object (sample size, gender, mean age, intervention and comparator, course of treatment, outcome measures, and follow-up time), and the extraction of the outcome indicators are continuous variable, and expressed as a mean and standard deviation, respectively. At the same time, the key factors of bias risk assessment are extracted.

### 2.6 Quality assessment/methodologic quality of included studies

The methodologic quality of systematic review reflects the risk of bias or validity in its process and results. Methodologic quality will be assessed based on the Cochrane Collaboration's tool (Cochrane Handbook 5.1.0). Two trained researchers (KS and AH) will independently evaluate the risk of bias of the included studies. In case of dispute, submit to corresponding author HZ for arbitration.

Cochrane bias risk assessment tool will be used to assess the risk of RCTs being included in NMA, including^[27]^: random sequence generation; allocation concealment; blinding of the subjects and researchers; blinding of outcome assessment; incomplete outcome data; selective reporting; and other bias.

### 2.7 Data synthesis and statistical methods

#### 2.7.1 Network meta-analysis

This study uses Aggregate Data Drug Information System 1.16.8 for NMA.^[28]^ Aggregate Data Drug Information System software uses Markov chain-Monte Carlo (MCMC) algorithm to for priori evaluation and processing the extracted data based on Bayesian framework, so as to provide support for further research and decision. Preset model parameters: 4 chains are used for simulation analysis, with an initial value of 2.5, a step size of 10, 20,000 annealing times, and 50,000 simulation iterations. Firstly, the network evidence plot is generated according to different outcome indicators, standardized mean differences or odds ratios is used as the effect quantity for statistical analysis, both with 95% confidence intervals. According to the results of the NMA, rank probability plot of various CAM therapies is generated and sorted by dominance, with Rank 1 being the optimal sort.

#### 2.7.2 Statistical model selection

Node-split model is used to verify the consistency of the corresponding data. If there is no statistical difference (*P* > .05) between direct comparison and indirect comparison, the consistency model is used, whereas the inconsistency model is used for analysis. If the consistency model is adopted, then the stability of the results is verified by the inconsistency model: when the inconsistency factors including 0, at the same time inconsistency standard deviation including 1 says the result of consistency model is more stable and reliable. At the same time, various analysis models are iterated with preset parameters, and the convergence of iteration effect is judged by potential scale-reduced factor (PSRF). When the PSRF value is close to or equal to 1 (1 ≤ PSRF ≤ 1.05), the convergence is complete, the model has good stability, and the conclusion of analysis is reliable. If the PSRF value is not in this range, the iteration continues manually until the PSRF value reaches the range standard.

#### 2.7.3 Heterogeneity test

Before the combination of effect size, the heterogeneity of the included literature is tested using Stata. When inter-study heterogeneity exists, the random effect model is used. For comparison of each pair, heterogeneity is assessed by the statistic *I*^2^ value. When *I*^2^ > 50%, it indicates that there is heterogeneity between studies, and the source of heterogeneity should be further searched. When *I*^2^ < 50%, interstudy heterogeneity is considered to be small or there is no obvious heterogeneity.

#### 2.7.4 Sensitivity analysis

If necessary, the sensitivity analysis will be used to assess the effect of each study on the random effects model. The sensitivity of the general combined effect of all outcome indicators is analyzed by the exclusion method. That is, each study is excluded, and the remaining studies will be reanalyzed to identify the stability of the results. If there is no qualitative change in the combined effect showed in the results, the results are stable.

#### 2.7.5 Subgroup analysis

If necessary, we will conduct a subgroup analysis of duration of treatment, age, history of PTSD, and research quality.

#### 2.7.6 Small sample effect/publication bias

If 10 or more studies are included in the NMA, a comparison-adjusted funnel plot is developed using Stata to evaluate the presence of small sample effects or publication bias in the intervention network. Descriptive analysis will be carried out through the symmetry of funnel plot. If the plot is asymmetric and there is no inverted funnel shape, it indicates that there may be publication bias. This may be related to the difficulty in the publication of the literature with negative results and the low quality of the inclusion methods.

#### 2.7.7 Dealing with missing data

If the required data are lost or incomplete, we will contact the corresponding author of the original document or the relevant email address of the first author. If there is no response, the record is excluded.

#### 2.7.8 Evaluating the quality of the evidence

To grade evidence quality and understand the current situation of evidence rating thereby analyzing possible problems, The Grading of Recommendations Assessment, Development and Evaluation (GRADE) instrumental will be used to assess the quality of evidence in the NMA.^[29]^ Based on the risk of bias, inconsistency, imprecision, indirection, and publication bias, GRADE grades evidence quality into 4 levels: high, medium, low, and very low.

## 3 Discussions

The PTSD is excessive physical and mental stress caused by a major traumatic event, such as natural disasters and man-made traumas, characterized by tardiness and persistence, or even lifelong onset. At present, the COVID-19 outbreak has been a continuing crisis for every member of society, and there is also the possibility of PTSD.^[30]^ There has been a growing number of studies on CAM therapies for PTSD in the late years, compared to the current first-line treatment for PTSD (psychotherapy and medication), CAM therapies have special strengths, it require less talking and disclosure than psychotherapy, and may not carry the risks of side effects from pharmaceutical approaches.^[17]^ However, there is no decision-making conclusion as to which CAM method to select in clinical practice.

Thus, our study employed a NMA of all RCTs of CAM therapies for PTSD, including acupuncture, moxibustion, Chinese herbal medicines, meditation, yoga, deep-breathing exercises, mind-body therapy, tai chi, etc, to synthesize all this evidence and perform an integrated rank of available CAM treatments for PTSD. We hope that the study results will encourage further suggestions for CAM clinical practice or guideline to a certain extent.

There are some potential limitations are predictable to this study. For example, different combination of acupoints and difference of methodologic quality in the trials may cause significant heterogeneity. In addition, due to the limitations of language ability, the authors only search for literature in English and Chinese, and may lead to the potential risk of ignoring essential literature.

## Author contributions

**Conceptualization:** Kai Song, Fanjie Xiong, Ning Ding.

**Data curation:** Kai Song, Fanjie Xiong, Ailing Huang.

**Formal analysis:** Fanjie Xiong, Ning Ding.

**Funding acquisition:** Ning Ding, Hong Zhang.

**Methodology:** Kai Song, Fanjie Xiong, Ailing Huang.

**Project administration:** Kai Song, Ailing Huang, Ning Ding.

**Writing – original draft**: Kai Song, Fanjie Xiong.

**Writing – review & editing:** Hong Zhang.
